# Supplementary material for: Heparin-based hydrogel scaffolding alters the transcriptomic profile and increases the chemoresistance of MDA-MB-231 triple-negative breast cancer cells
Source: Biomater Sci. 2020 Feb 13;8(10):2786–96. doi: 10.1039/c9bm01481k (PMC7497406; doi:10.1039/c9bm01481k)
Supplement: Supplementary file 2 [file BM-008-C9BM01481K-s002.zip › Supplementary File 4/HP-BhydrogelvControl/Pathways/my_analysis.Gsea.1545201079907/HALLMARK_UNFOLDED_PROTEIN_RESPONSE.html]

Details for gene set HALLMARK\_UNFOLDED\_PROTEIN\_RESPONSE[GSEA]

|  || Dataset | expr.class.cls#HYD\_versus\_CONTROL.class.cls#HYD\_versus\_CONTROL\_repos |
| Phenotype | class.cls#HYD\_versus\_CONTROL\_repos |
| Upregulated in class | CONTROL |
| GeneSet | HALLMARK\_UNFOLDED\_PROTEIN\_RESPONSE |
| Enrichment Score (ES) | -0.27861845 |
| Normalized Enrichment Score (NES) | -1.2538443 |
| Nominal p-value | 0.073469386 |
| FDR q-value | 0.107810415 |
| FWER p-Value | 0.623 |
Table: GSEA Results Summary

  

Fig 1: Enrichment plot: HALLMARK\_UNFOLDED\_PROTEIN\_RESPONSE      
 Profile of the Running ES Score & Positions of GeneSet Members on the Rank Ordered List

  

| PROBE | DESCRIPTION (from dataset) | GENE SYMBOL | GENE\_TITLE | RANK IN GENE LIST | RANK METRIC SCORE | RUNNING ES | CORE ENRICHMENT || 1 | STC2 | na |  |  | 313 | 2.647 | 0.0197 | No |
| 2 | ARFGAP1 | na |  |  | 506 | 2.333 | 0.0414 | No |
| 3 | ZBTB17 | na |  |  | 626 | 2.178 | 0.0648 | No |
| 4 | EIF4A1 | na |  |  | 1639 | 1.516 | 0.0325 | No |
| 5 | RRP9 | na |  |  | 1722 | 1.481 | 0.0484 | No |
| 6 | CXXC1 | na |  |  | 2221 | 1.265 | 0.0395 | No |
| 7 | ATF3 | na |  |  | 2583 | 1.150 | 0.0363 | No |
| 8 | SHC1 | na |  |  | 3004 | 1.036 | 0.0284 | No |
| 9 | TATDN2 | na |  |  | 3411 | 0.935 | 0.0199 | No |
| 10 | KHSRP | na |  |  | 3808 | 0.850 | 0.0107 | No |
| 11 | EXOSC4 | na |  |  | 3894 | 0.834 | 0.0176 | No |
| 12 | NOP14 | na |  |  | 3978 | 0.816 | 0.0244 | No |
| 13 | GOSR2 | na |  |  | 4027 | 0.806 | 0.0329 | No |
| 14 | EDC4 | na |  |  | 4479 | 0.720 | 0.0191 | No |
| 15 | ATF4 | na |  |  | 4526 | 0.707 | 0.0263 | No |
| 16 | PDIA5 | na |  |  | 4617 | 0.691 | 0.0310 | No |
| 17 | NOP56 | na |  |  | 4859 | 0.647 | 0.0272 | No |
| 18 | GEMIN4 | na |  |  | 4888 | 0.644 | 0.0345 | No |
| 19 | TSPYL2 | na |  |  | 4991 | 0.628 | 0.0377 | No |
| 20 | SLC7A5 | na |  |  | 5187 | 0.602 | 0.0357 | No |
| 21 | DCTN1 | na |  |  | 5912 | 0.498 | 0.0046 | No |
| 22 | ERN1 | na |  |  | 5950 | 0.493 | 0.0094 | No |
| 23 | EXOSC2 | na |  |  | 6314 | 0.442 | -0.0036 | No |
| 24 | EIF4A3 | na |  |  | 6334 | 0.439 | 0.0014 | No |
| 25 | SLC1A4 | na |  |  | 6805 | 0.382 | -0.0180 | No |
| 26 | CEBPB | na |  |  | 6839 | 0.379 | -0.0146 | No |
| 27 | EXOSC10 | na |  |  | 7002 | 0.360 | -0.0182 | No |
| 28 | EXOSC9 | na |  |  | 7224 | 0.336 | -0.0252 | No |
| 29 | LSM1 | na |  |  | 7285 | 0.329 | -0.0238 | No |
| 30 | EIF2AK3 | na |  |  | 7881 | 0.264 | -0.0514 | No |
| 31 | PREB | na |  |  | 7964 | 0.253 | -0.0523 | No |
| 32 | HYOU1 | na |  |  | 8209 | 0.231 | -0.0619 | No |
| 33 | NFYA | na |  |  | 8318 | 0.218 | -0.0646 | No |
| 34 | H2AFX | na |  |  | 8328 | 0.216 | -0.0621 | No |
| 35 | WFS1 | na |  |  | 8337 | 0.215 | -0.0596 | No |
| 36 | NFYB | na |  |  | 8358 | 0.212 | -0.0578 | No |
| 37 | LSM4 | na |  |  | 9131 | 0.136 | -0.0963 | No |
| 38 | NOLC1 | na |  |  | 9142 | 0.135 | -0.0950 | No |
| 39 | EXOSC1 | na |  |  | 9193 | 0.126 | -0.0959 | No |
| 40 | FUS | na |  |  | 9228 | 0.125 | -0.0960 | No |
| 41 | XBP1 | na |  |  | 9718 | 0.073 | -0.1206 | No |
| 42 | EXOSC5 | na |  |  | 10572 | -0.013 | -0.1651 | No |
| 43 | DCP1A | na |  |  | 11038 | -0.071 | -0.1885 | No |
| 44 | IGFBP1 | na |  |  | 11345 | -0.095 | -0.2032 | No |
| 45 | CEBPG | na |  |  | 11400 | -0.101 | -0.2047 | No |
| 46 | YIF1A | na |  |  | 11570 | -0.122 | -0.2119 | No |
| 47 | DKC1 | na |  |  | 11919 | -0.166 | -0.2278 | No |
| 48 | ATP6V0D1 | na |  |  | 12163 | -0.194 | -0.2379 | No |
| 49 | DNAJA4 | na |  |  | 12228 | -0.203 | -0.2385 | No |
| 50 | DDX10 | na |  |  | 12344 | -0.220 | -0.2415 | No |
| 51 | SKIV2L2 | na |  |  | 12351 | -0.220 | -0.2388 | No |
| 52 | CCL2 | na |  |  | 12375 | -0.222 | -0.2370 | No |
| 53 | TARS | na |  |  | 12761 | -0.254 | -0.2537 | No |
| 54 | BAG3 | na |  |  | 12765 | -0.254 | -0.2504 | No |
| 55 | CALR | na |  |  | 12797 | -0.257 | -0.2485 | No |
| 56 | PARN | na |  |  | 12980 | -0.280 | -0.2542 | No |
| 57 | CHAC1 | na |  |  | 13123 | -0.302 | -0.2576 | No |
| 58 | WIPI1 | na |  |  | 13158 | -0.308 | -0.2552 | No |
| 59 | EIF4EBP1 | na |  |  | 13164 | -0.309 | -0.2512 | No |
| 60 | CNOT4 | na |  |  | 13269 | -0.319 | -0.2523 | No |
| 61 | PAIP1 | na |  |  | 13339 | -0.328 | -0.2515 | No |
| 62 | SDAD1 | na |  |  | 13459 | -0.346 | -0.2530 | No |
| 63 | KIF5B | na |  |  | 13478 | -0.350 | -0.2491 | No |
| 64 | FKBP14 | na |  |  | 13619 | -0.373 | -0.2514 | No |
| 65 | EIF4A2 | na |  |  | 13623 | -0.374 | -0.2465 | No |
| 66 | DCP2 | na |  |  | 13724 | -0.391 | -0.2464 | No |
| 67 | HSPA5 | na |  |  | 13730 | -0.393 | -0.2413 | No |
| 68 | IARS | na |  |  | 13999 | -0.416 | -0.2497 | No |
| 69 | TUBB2A | na |  |  | 14107 | -0.429 | -0.2494 | No |
| 70 | BANF1 | na |  |  | 14255 | -0.454 | -0.2509 | No |
| 71 | SLC30A5 | na |  |  | 14356 | -0.471 | -0.2498 | No |
| 72 | DDIT4 | na |  |  | 14408 | -0.478 | -0.2459 | No |
| 73 | PSAT1 | na |  |  | 14610 | -0.508 | -0.2495 | No |
| 74 | NHP2 | na |  |  | 14692 | -0.521 | -0.2466 | No |
| 75 | HERPUD1 | na |  |  | 15077 | -0.574 | -0.2589 | No |
| 76 | XPOT | na |  |  | 15237 | -0.603 | -0.2590 | No |
| 77 | EXOC2 | na |  |  | 15260 | -0.610 | -0.2519 | No |
| 78 | CNOT2 | na |  |  | 15417 | -0.636 | -0.2514 | No |
| 79 | CNOT6 | na |  |  | 15938 | -0.731 | -0.2687 | Yes |
| 80 | SRPRB | na |  |  | 15991 | -0.742 | -0.2613 | Yes |
| 81 | POP4 | na |  |  | 16007 | -0.747 | -0.2519 | Yes |
| 82 | SEC31A | na |  |  | 16038 | -0.756 | -0.2432 | Yes |
| 83 | EIF2S1 | na |  |  | 16087 | -0.767 | -0.2352 | Yes |
| 84 | PDIA6 | na |  |  | 16357 | -0.823 | -0.2381 | Yes |
| 85 | RPS14 | na |  |  | 16474 | -0.848 | -0.2326 | Yes |
| 86 | EDEM1 | na |  |  | 16664 | -0.889 | -0.2304 | Yes |
| 87 | TTC37 | na |  |  | 16699 | -0.898 | -0.2199 | Yes |
| 88 | MTHFD2 | na |  |  | 16986 | -0.982 | -0.2215 | Yes |
| 89 | CKS1B | na |  |  | 17139 | -1.027 | -0.2155 | Yes |
| 90 | IFIT1 | na |  |  | 17218 | -1.058 | -0.2052 | Yes |
| 91 | DNAJB9 | na |  |  | 17375 | -1.111 | -0.1982 | Yes |
| 92 | HSPA9 | na |  |  | 17460 | -1.137 | -0.1871 | Yes |
| 93 | SERP1 | na |  |  | 17632 | -1.213 | -0.1796 | Yes |
| 94 | SEC11A | na |  |  | 17640 | -1.217 | -0.1633 | Yes |
| 95 | EIF4E | na |  |  | 17756 | -1.264 | -0.1521 | Yes |
| 96 | SPCS3 | na |  |  | 17860 | -1.315 | -0.1396 | Yes |
| 97 | HSP90B1 | na |  |  | 18044 | -1.410 | -0.1300 | Yes |
| 98 | ATF6 | na |  |  | 18108 | -1.448 | -0.1136 | Yes |
| 99 | ASNS | na |  |  | 18216 | -1.509 | -0.0986 | Yes |
| 100 | ALDH18A1 | na |  |  | 18253 | -1.533 | -0.0796 | Yes |
| 101 | KDELR3 | na |  |  | 18309 | -1.571 | -0.0611 | Yes |
| 102 | SSR1 | na |  |  | 18609 | -1.786 | -0.0524 | Yes |
| 103 | IMP3 | na |  |  | 18692 | -1.889 | -0.0309 | Yes |
| 104 | SPCS1 | na |  |  | 18745 | -1.947 | -0.0071 | Yes |
| 105 | DNAJC3 | na |  |  | 18949 | -2.261 | 0.0130 | Yes |
Table: GSEA details [plain text format]

  

Fig 2: HALLMARK\_UNFOLDED\_PROTEIN\_RESPONSE      
 Blue-Pink O' Gram in the Space of the Analyzed GeneSet

  

Fig 3: HALLMARK\_UNFOLDED\_PROTEIN\_RESPONSE: Random ES distribution      
 Gene set null distribution of ES for **HALLMARK\_UNFOLDED\_PROTEIN\_RESPONSE**
